# Genetic alterations that deregulate RB and PDGFRA signaling pathways drive tumor progression in *IDH2*-mutant astrocytoma

**DOI:** 10.1186/s40478-023-01683-x

**Published:** 2023-11-27

**Authors:** Kensuke Tateishi, Yohei Miyake, Taishi Nakamura, Hiromichi Iwashita, Takahiro Hayashi, Akito Oshima, Hirokuni Honma, Hiroaki Hayashi, Kyoka Sugino, Miyui Kato, Kaishi Satomi, Satoshi Fujii, Takashi Komori, Tetsuya Yamamoto, Daniel P. Cahill, Hiroaki Wakimoto

**Affiliations:** 1 Department of Neurosurgery, Graduate School of Medicine, Yokohama City University, 3-9 Fukuura, Kanazawa, Yokohama, 2360004 Japan; 2 Laboratory of Biopharmaceutical and Regenerative Science, Graduate School of Medical Science, Yokohama City University, Yokohama, Japan; 3 Neurosurgical-Oncology Laboratory, Yokohama City University, Yokohama, Japan; 4 Department of Pathology, Yokohama City University Hospital, Yokohama, Japan; 5 Department of Diagnostic Pathology, Yokohama City University Hospital, Yokohama, Japan; 6 Department of Pediatrics, Graduate School of Medicine, Yokohama City University, Yokohama, Japan; 7 Department of Pathology, Kyorin University School of Medicine, Tokyo, Japan; 8 Department of Molecular Pathology, Graduate School of Medicine, Yokohama City University, Yokohama, Japan; 9 Department of Laboratory Medicine and Pathology (Neuropathology), Tokyo Metropolitan Neurological Hospital, Tokyo, Japan; 10 Department of Neurosurgery, Massachusetts General Hospital, Boston, MA USA; 11 Translational-Neurooncology Laboratory, Brain Tumor Research Center, Massachusetts General Hospital/Harvard Medical School, Boston, MA USA

**Keywords:** IDH2 mutation, Astrocytoma, Malignant phenotype, PDX

## Abstract

In *IDH*-mutant astrocytoma, *IDH2* mutation is quite rare and biological mechanisms underlying tumor progression in *IDH2*-mutant astrocytoma remain elusive. Here, we report a unique case of *IDH2* mutant astrocytoma, CNS WHO grade 3 that developed tumor progression. We performed a comprehensive genomic and epigenomic analysis for primary and recurrent tumors and found that both tumors harbored recurrent *IDH2*^*R172K*^ and *TP53*^*R248W*^ mutation with *CDKN2A/B* hemizygous deletion. We also found amplifications of *CDK4* and *MDM2* with *PDGFRA* gain in the recurrent tumor and upregulated protein expressions of these genes. We further developed, for the first time, a xenograft mouse model of *IDH2*^*R172K*^ and *TP53*^*R248W*^ mutant astrocytoma from the recurrent tumor, but not from the primary tumor. Consistent with parent recurrent tumor cells, amplifications of *CDK4* and *MDM2* and *PDGFRA* gain were found, while *CDKN2A/B* was identified as homozygous deletion in the xenografts, qualifying for integrated diagnosis of astrocytoma, IDH2-mutant, CNS WHO grade 4. Cell viability assay found that CDK4/6 inhibitor and PDGFR inhibitor potently decreased cell viability in recurrent tumor cells, as compared to primary tumor cells. These findings suggest that gene alterations that activate retinoblastoma (RB) signaling pathways and PDGFR may drive tumor progression and xenograft formation in *IDH2-*mutant astrocytoma, which is equivalent to progressive *IDH1*-mutant astrocytoma. Also, our findings suggest that these genomic alterations may represent therapeutic targets in *IDH2*-mutant astrocytoma.

**Supplementary Information:**

The online version contains supplementary material available at 10.1186/s40478-023-01683-x.

## Introduction

Since the discovery of *IDH1* mutation in gliomas [30], *IDH1* mutation has been considered one of the most fundamental genetic alterations in diffuse lower-grade gliomas (LGGs). In *IDH*-mutant gliomas, the vast majority of mutations are at codon 132 of *IDH1* and mostly heterozygous substitution from arginine to histidine (R132H) [35, 46]. Astrocytoma typically harbors *IDH1*, *TP53*, and *ATRX* mutations [4, 20]. In general, *IDH1-*mutant astrocytomas have better prognosis than *IDH1/2*-wildtype gliomas. However, most *IDH1*-mutant astrocytomas eventually develop a malignant phenotype [25]. Several studies have uncovered molecular mechanisms of malignant transformation in *IDH1*-mutant astrocytoma [5, 33, 37, 42]. For instance, we have demonstrated that additional “tertiary mutations”, such as *PDGFRA* and *MYCN* amplification, promoted patient tumor progression and xenograft formation in *IDH1*-mutant astrocytoma. We also found that xenograft formation was correlated with poor prognosis in *IDH1*-mutant astrocytoma patients [42]. Using genetically engineered mouse models, Philip et al. reported that IDH1^R132H^ cooperated with PDGFA and loss of *Cdkn2a*, *Atrx*, and *Pten* to promote high-grade astrocytoma in vivo [31]. These data indicate that acquired pathogenic gene alterations promote tumor progression in *IDH1-*mutant astrocytoma, resulting in dismal outcomes.

In mammalian cells, IDH1 is located in the cytoplasm, while IDH2 is in the mitochondria. *IDH2* mutation is commonly found in acute myeloid leukemia [45]. On the other hand, *IDH2*^*R172*^ mutation was also identified in astrocytoma, with less than 3% frequency [9]. Another study analyzed 811 glioma samples and identified only 3 of 266 (1.1%) astrocytomas harbored *IDH2*-mutation [44]. These findings suggest that *IDH2*-mutation is quite rare in astrocytoma. Both mutant *IDH1* and *IDH2* inhibit this enzymatic activity and instead produce 2-hydroxyglutarate (2-HG) from α-KG. 2-HG induces the global DNA and histone methylation phenotype by blocking α-KG dependent dioxygenases and promote gliomagenesis [12, 14, 29]. Therefore, *IDH2* mutation is considered as equivalent to *IDH1* mutation and *IDH2*-mutant gliomas are conventionally analyzed along with *IDH1*-mutant gliomas. However, the molecular mechanisms of malignant progression are poorly understood in *IDH2*-mutant astrocytoma. Additionally, since *IDH2-*mutant glioma xenograft model is lacking, translational insight is scant to date. Here, we report a patient with *IDH2*^*R172K*^-mutant astrocytoma that progressed during follow-up. We comprehensively performed genomic and epigenomic analyses for both the initial and recurrent tumors. We further developed the first *IDH2*^*R172K*^ mutant astrocytoma, CNS WHO grade 4 xenograft mouse model from recurrent tumor. Our data reveal a molecular mechanism of how genomic alterations promote tumor progression and xenograft formation in *IDH2*-mutant astrocytoma.

## Material and methods

### Creation of primary cultured cells

Fresh tumor specimens were obtained from surgery and enzymatically dissociated with 0.1% of Trypsin and DNase. Primary cultured cells were maintained in serum-free neural stem cell medium (Neurobasal Medium, Gibco), supplemented with L-glutamine (Gibco), B27 (Gibco), N2 (Gibco), human recombinant EGF (R&D Systems), human FGF-basic (Alomone Labs), and Antibiotic–Antimycotic (Gibco) as previously described [41]. Dissociated cells were cryopreserved and used for in vitro experiments.

### Cell viability analysis

To assess cell viability, tumor cells were dissociated into single cells and seeded into 96-well plates at 3000 cells/well. After 6 h, chemical inhibitors were serially diluted and added to wells. Cell viability was measured by CellTiter-Glo (Promega) assay at day 3. The relative cell viability was indicated as the percentage viability of the DMSO control. Abemaciclib (Selleck), AC710 (Tocris Bioscience), Enasidenib (AG-221, MedChemExpress), GDC-0068 (Cayman), LY294002 (Sellek), Palbociclib (Toronto Research chemicals), and Tyrphostin A9 (Focus Biomolecules) were used.

### Xenograft models

1 × 10^5^ Cells were orthotopically implanted into the right striatum of 4–6 week-old female SCID Beige mice (Charles River, Yokohama) within 12 h after dissociation. Mice were monitored 2–3 times per week and sacrificed when neurologic deficits or general conditions reached the criteria for euthanasia. Brains were harvested and used for pathological and genomic evaluation. All mouse experiments were approved by the Institutional Animal Care and Use Committee at YCU (IRB No. FA22-011).

### Western blotting

Cells were lysed in RIPA buffer (Sigma-Aldrich) with protease inhibitor cocktail tablets (Roche). Fifty μg of protein was separated by 10% SDS-PAGE and transferred to polyvinylidene difluoride membranes (Millipore) by electroblotting. After blocking with Bullet Blocking One for western blotting (Nacalai Tesque), the membranes were incubated with primary antibodies at 4 °C overnight. After washing and incubation with horseradish peroxidase–conjugated secondary antibodies (CST), the blots were washed, and the signals were visualized with chemiluminescent HRP substrate (Merck Millipore). The primary antibodies used were cleaved-PARP ([diluted 1:1000], GeneTex, Cat. #GTX132329), GAPDH ([diluted 1:4000], GeneTex, Cat. #GTX100118), IDH2^R172K^ ([diluted 1:500], Medical and Biological laboratories, MBL, Cat. #D328-3), H3K27me3 ([diluted 1:5000], Cell Signaling Technology, CST, Cat. #9733 T), H3K9me3 ([diluted 1:5000], Abcam, Cat. #ab8898), Histone H3 antibody ([diluted 1:5000], Abcam, Cat. #ab1791), phospho-AKT ([diluted 1:1000], GeneTex, Cat. #GTX128414), phospho-ERK ([diluted 1:1000], Bethyl Laboratories, Cat. #A303-608A-M), phospho-MEK ([diluted 1:1000], CST, Cat. #9154S), and phospho-mTOR ([diluted 1:500], Merck, Cat. #09-213). Western blotting images were evaluated qualitatively.

### Histopathological analysis

Tumor tissue specimens were fixed in 10% neutral-buffered formalin and embedded in paraffin. Hematoxylin and eosin staining was performed using standard procedures. For immunohistochemistry, 5-µm thick sections were deparaffinized, treated with 0.5% H_2_O_2_ in methanol, rehydrated, and heated in a microwave for 20 min for antigen retrieval. After blocking with serum, tissue sections were incubated with primary antibodies against Akt ([diluted 1:1000], CST, Cat. #4691), CDK4 ([diluted 1:1000], Bioss Antibodies, Cat. #BS-0633R), ERK ([diluted 1:1000], CST, Cat. #4695), Ki-67 ([diluted 1:1000], Novus Biologicals, Cat. #NB600-1252), MDM2 ([diluted 1:1000], GeneTex, Cat. #GTX100531), p53 ([diluted 1:1000], Novus Biologicals, Cat. #NBP2-44982), phospho-AKT ([diluted 1:1000], GeneTex, Cat. #GTX128414), phospho-ERK ([diluted 1:1000], Bethyl Laboratories, Cat. #A303-608A), phospho-PDGFRA ([diluted 1:1000], CST, Cat. #3170 T), and phospho-Rb ([diluted 1:1000], CST, Cat. #8516S) at 4 °C overnight. The next day, the sections were washed with phosphate-buffered saline, incubated with biotinylated secondary antibodies for 30 min at room temperature, and then incubated with ABC solution (PK-6101, PK-6102; Vector Laboratories) for 30 min. Finally, the sections were incubated with DAB (K3467, Dako) and counterstained with hematoxylin. Three images per each specimen were obtained for quantitative analysis. Cells positive for phosphorylated proteins were evaluated quantitatively. Only strongly stained cells were considered positive.

### Genomic analysis

Genomic DNA was extracted using Dneasy Blood & Tissue (Qiagen), according to the manufacturer’s protocol. To evaluate single nucleotide variants (SNVs), insertion/deletion, and copy number alterations (CNAs), whole exome sequencing (WES) was performed as previously described [38]. Somatic SNV was detected by MuTect, while the somatic InDel was identified by Strelka. Control-FREEC was used to detect somatic CNV. ANNOVAR was used to perform variant annotation. dbSNP, COSMIC, OMIM, GWAS Catalog, and HGMD were used to find reported information of the variants [23]. The multiplex polymerase chain reaction (PCR) technology (MGH SNaPShot) was also performed for validation, as previously described [39]. *IDH1*^*R132H*^ and *IDH2*^*R172K*^*, TP53*^*R248W*^, and *TERT* promoter SNVs were also assessed by Sanger sequencing. Primer sequences for Sanger sequencing are listed in the Additional file 2: Table S1. *CDK4*, *CDKN2A*, *EGFR*, *MDM2*, *PDGFRA*, *PTEN*, and *TP53* CNAs were selectively assessed using multiplex ligation-dependent probe amplification (MLPA), according to the manufacturer’s instructions (SALSA MLPA KIT probe mix P105-D3, MRC-Holland). SALSA MLPA KIT probe mix P088-C2 was used to validate chromosome 1p and/19q and *CDKN2A* status. The MLPA data were collected using an ABI 3730xL Genetic Analyzer (FASMAC, Japan) and analyzed using Coffalyzer.Net Software (MRC-Holland). The copy number status was defined using the following thresholds: homozygous deletion (HD, x < 0.4), hemizygous deletion (0.4 < x < 0.7), gain (1.3 < x < 2.0), and amplification (x > 2.0), according to previous studies [17].

### DNA methylation array analysis

The Infinium MethylationEPIC v.1.0 BeadChip Kit (Illumina) was used to obtain genome-wide DNA methylation profiles and copy number alterations. The detailed protocol has been described previously [16]. The cut-off value for amplification (0.35) and homozygous deletion ( − 0.415) was used [37].

### Statistical analysis

Statistical analysis was performed using JMP Pro17.0.0 software (Cary, NC) and GraphPad Prism (ver. 10.0.3, San Diego, CA). For parametric analysis, a two-tailed *t*-tests was used. Survival analysis using datasets was performed by Kaplan–Meier method, and the log-rank test was used to compare survival differences. The data were expressed as the mean ± SEM. *P*-value < 0.05 was considered as statistically significant.

## Results

### Case presentation

**Fig. 1:**
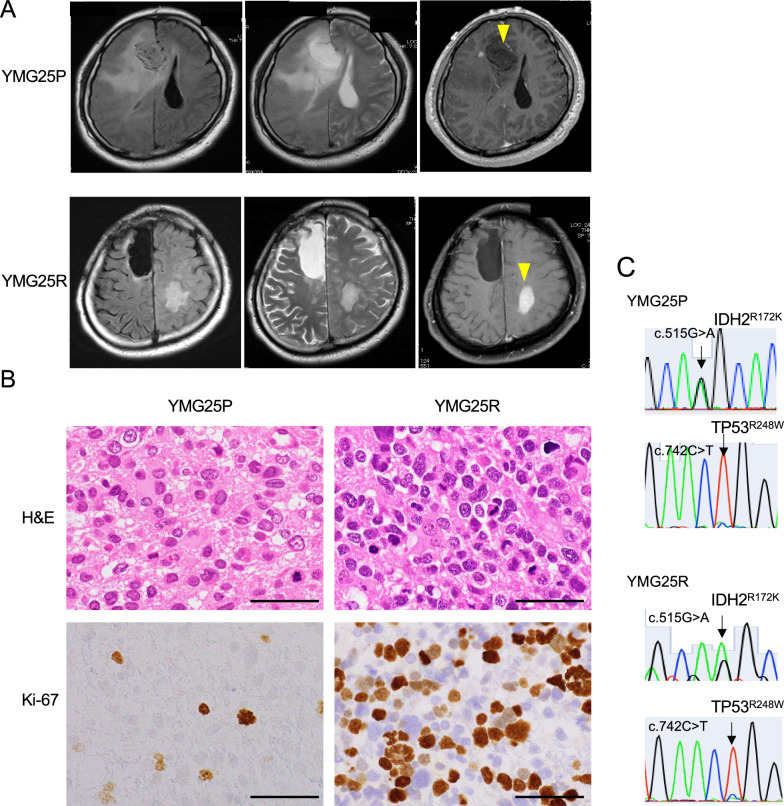
Clinical characteristics of *IDH2*-mutant astrocytoma patient. **A** Magnetic resonance imaging showing FLAIR (left), T2-weighted (middle), and gadolinium-enhanced T1-weighted (right) images for initial tumor (YMG25P, upper panel) and recurrent tumor (YMG25R, lower panel). **B** Hematoxylin and eosin staining (upper) and Ki-67 immunohistochemistry (lower) for YMG25P (left) and YMG25R (right) tumors. Bars, 50 μm. **C** Sanger sequencing indicating *IDH2* (c.515G > A, R172K) and *TP53* (c.742C > T, R248W) mutation in YMG25P (upper) and YMG25R (lower)

A 44-year-old man complained a headache. Magnetic resonance imaging (MRI) demonstrated a non-enhancing tumor with surrounding edema in the right frontal lobe. The T2/FLAIR mismatch sign was observed (Fig. 1A, upper panels). ^18^F-fluorodeoxyglucose (FDG)-PET demonstrated a lower uptake in the tumor, compared with the contralateral cerebral hemisphere. ^11^C-methionnine (MET)-PET revealed a weak uptake of the tumor (maximum standardized uptake value; 2.0, Additional file 1: Fig. S1A). We performed subtotal tumor resection at the right superior frontal gyrus (YMG25P). Hematoxylin and eosin staining showed high cellular proliferation of astrocytic cells with 4 mitotic figures per mm^2^. Nuclear atypia was observed, while microvascular proliferation and necrosis were absent (Fig. 1B). Immunostaining for p53 was positive, whereas IDH1^R132H^ was negative and ATRX was lost (Additional file 1: Fig. S1B). Pathogenic mutations of *IDH2*^*R172K*^ and *TP53*^*R248W*^ were identified, (Fig. 1C, Additional file 2: Tables S2 and S3). On the other hand, *ATRX* and *TERT* promoter SNV were not identified.

Postoperatively, the patient received radiotherapy (60 Gy/30 fractions) with concomitant temozolomide (TMZ), and was subsequently treated with TMZ for 24 cycles. However, 32 months after the initial diagnosis, MRI demonstrated a contrast enhancing tumor in the contralateral left parietal lobe (Fig. 1A, lower panels). T2/FLAIR imaging showed a high signal intensity mass lesion, which was discontinuous from the initial tumor. We performed gross total resection of this tumor (YMG25R). Hematoxylin and eosin staining demonstrated high cellularity with 20 mitotic figures per mm^2^ in the recurrent tumor, but necrosis and microvascular proliferation were scant. The Ki-67 labeling index was 26% in YMG25R, which was relatively higher than that of YMG25P (11%, Fig. 1B). Immunostaining for p53 was positive, whereas IDH1^R132H^ was negative and ATRX was lost in YMG25R (Additional file 1: Fig. S1B), consistent with YMG25P. Genomic sequencing revealed the same *IDH2*^*R172K*^ and *TP53*^*R248W*^ heterozygous mutations (Fig. 1C). An elevated tumor mutation burden was found (23 mutations/Mb) in YMG25R, but additional pathogenic mutation was not identified (Additional file 2: Tables S2 and S3). After the second surgery, the patient received chemotherapy with procarbazine, nimustine, and vincristine. However, MRI showed progressive disease and bevacizumab was additionally administrated. Forty-eight months after initial diagnosis, the patient passed away due to tumor progression.

**Fig. 2:**
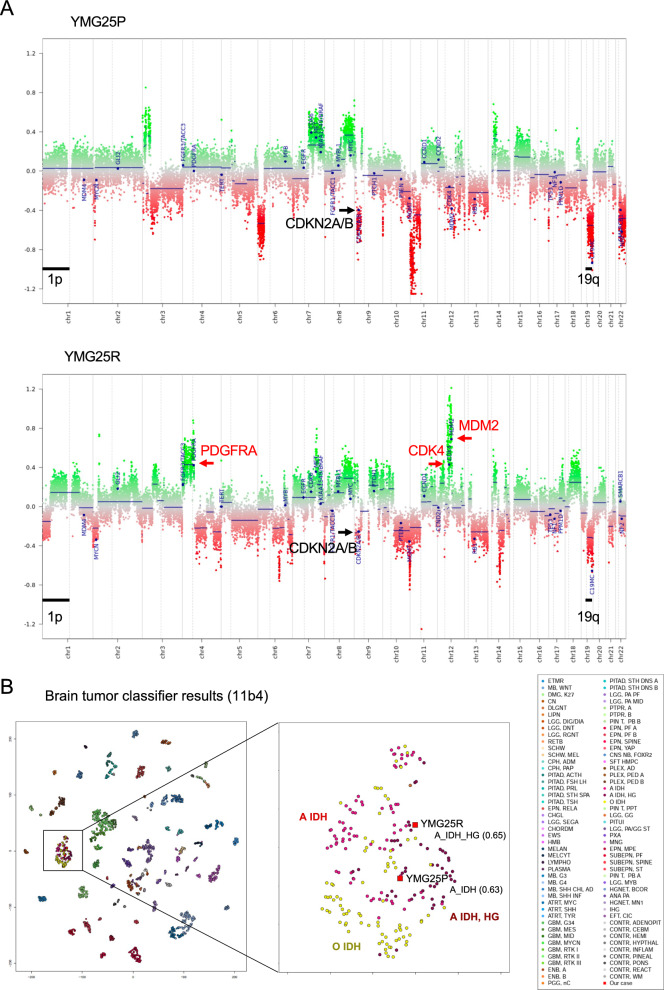
DNA methylation array-based tumor characteristics. **A** Copy number profiling of YMG25P (upper) and YMG25R (lower) tumor. **B** Unsupervised clustering using t-SNE analysis for initial tumor (YMG25P) and recurrent tumor (YMG25R). Brain tumor classifier was determined by version 11b4

Genome-wide DNA methylation array and MLPA revealed *CDKN2A* hemizygous deletion in YMG25R that was unchanged from YMG25P (Fig. 2A, Additional file 1: Fig. S2A). Also, partial deletion of chromosome 19 was found in YMG25P, while chromosome 1p and 19q partial deletion was observed in YMG25R (Fig. 2A, Additional file 1: Fig. S2B), which was described previously [28]. In YMG25P and YMG25R, methylation classifier results (version 11b4) indicated a classification matched to diffuse glioma, IDH mutant (Additional file 2: Table S4). Unsupervised clustering using t-SNE analysis, as indicated by DNA methylation analysis, demonstrated that both tumors were plotted close to astrocytoma, IDH-mutant (Fig. 2B). Methylation classifier (11b4) indicated subclass astrocytoma in YMG25P (score 0.63), and subclass high-grade astrocytoma in YMG25R (score 0.65, Fig. 2B, Additional file 2: Table S4). On the other hand, the newest version 12.8 matched YMG25P as diffuse glioma, IDH mutant and 1p/19q co-deleted in YMG25P (score 0.91), but did not match YMG25R to any classification (Additional file 2: Table S5). Since chromosome 1p/19q co-deletion, one of the essential criteria of “oligodendroglioma, IDH-mutant and 1p/19q-codeleted”, was absent in both tumors, the results of the Classifier version 12.8 were discordant with the molecular diagnosis. Collectively, the integrated diagnosis of YMG25P and YMG25R was astrocytoma, IDH-mutant, CNS WHO grade 3. *MGMT* promoter was methylated in both tumors (MGMT-STP27, Additional file 1: Fig. S2C). The reason for the discordance between the results of version 11b4 and 12.8 is unknown and has not been provided on the DKFZ website.

**Fig. 3:**
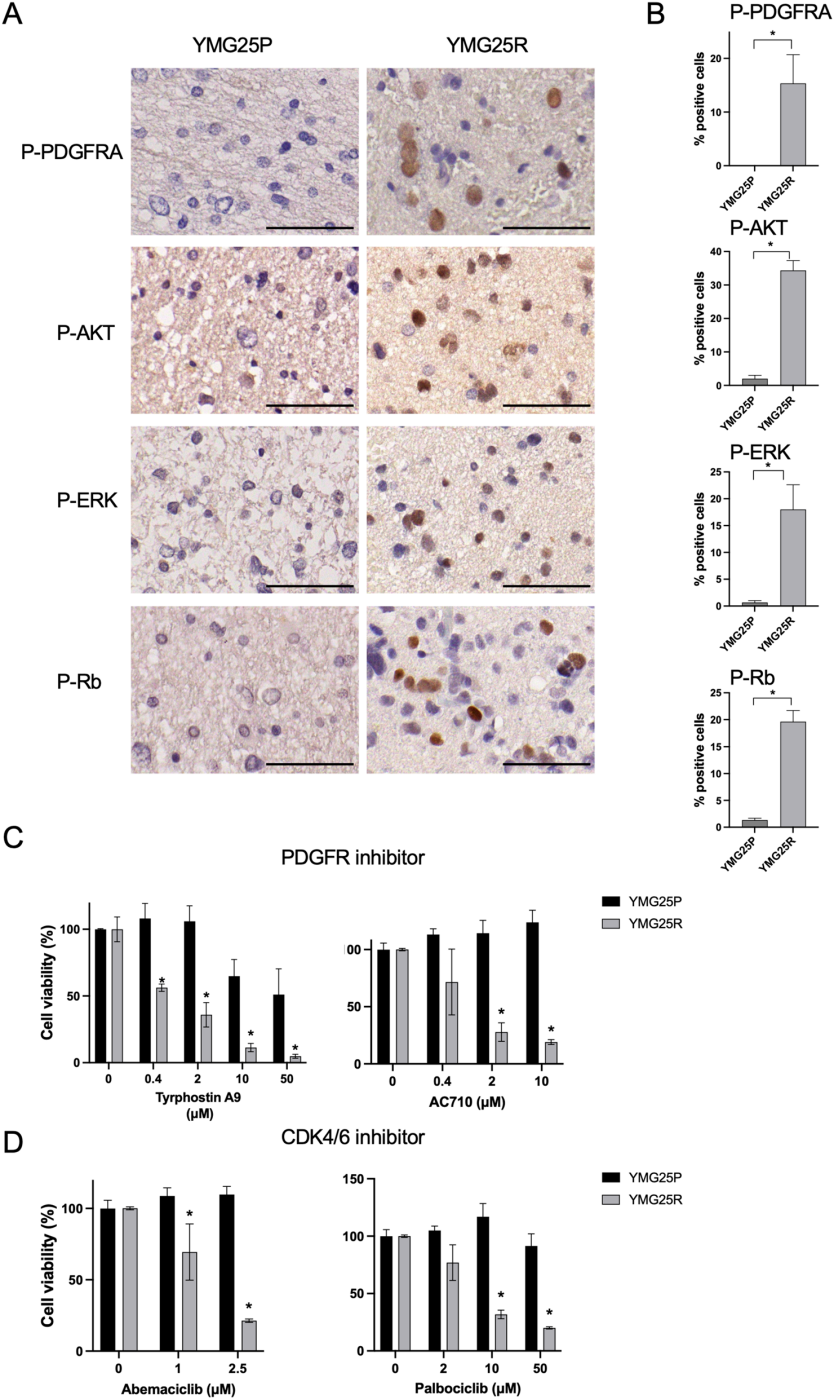
Protein expression and drug sensitivity for associated protein inhibitors. **A** Immunohistochemistry of indicated proteins for initial tumor (YMG25P, left) and recurrent tumor (YMG25R, right). Bars, 50 µm. **B** Bar graphs indicating % positive stained cells for indicated proteins. **C, D** Relative cell viability following PDGFR inhibitor (**C**) and CDK4/6 inhibitor (**D**) treatment at day3. DMSO, control. *P < 0.05. Data are represented as the mean ± SEM

Notably, amplifications of *CDK4* and *MDM2* and gain of *PDGFRA*, together with chromosome 4p gain were identified as newly acquired CNAs in YMG25R, as compared to the initial tumor YMG25P (Fig. 2A, Additional file 1: Fig. S2A). To assess differences of signaling pathway activation, we performed immunohistochemistry and western blot for phospho-PDGFRA, -AKT, -mTOR, -MEK, and -ERK, comparing YMG25P and YMG25R in tissue and cells derived. We found that the expression levels of these phospho-proteins were higher in YMG25R compared to YMG25P (Fig. 3A-B, Additional file 1: Fig. S3A-B). We also found that CDK4, MDM2, and phospho-Rb were upregulated in YMG25R as compared to YMG25P (Fig. 3A, Additional file 1: Fig. 3A).

We established patient-derived cultures from YMG25P and YMG25R, and tested response to targeted agents. Interestingly, we found a significantly increased sensitivity to PDGFR inhibitors (Tyrphostin A9 and AC710) in YMG25R cells compared with YMG25P cells (Fig. 3C). No difference was observed in response to treatment with PI3K inhibitor (LY294002) and AKT inhibitor (GDC-0068, Additional file 1: Fig. S3C). We also found that CDK4/6 inhibitors (Abemaciclib and Palbociclib) significantly decreased cell viability in YMG25R cells as compared with YMG25P cells (Fig. 3D). Previous clinical and preclinical studies have demonstrated that AML cells with *IDH2* mutation were highly sensitive to mutant IDH2 specific inhibitors [13, 43, 48]. To examine the potential impact of mutant IDH2 specific inhibitor on our *IDH2* mutant glioma cells, we performed cell viability assay and western blots. However, we did not find decreased cell viability or histone change in IDH2 inhibitor (AG-221)-treated YMG25R cells (Additional file 1: Fig. S3D–F).

**Fig. 4:**
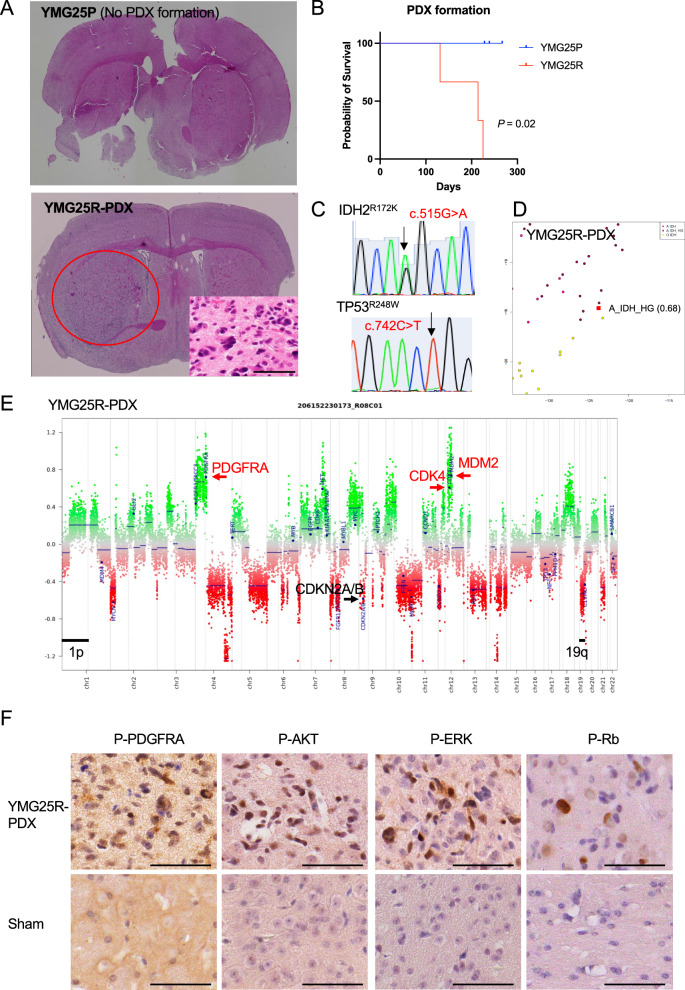
*IDH2*-mutant astrocytoma xenograft model. **A** Hematoxylin and eosin staining of non-xenograft formed mouse brain (YMG25P, upper) and xenograft formed mouse brain (YMG25R, lower). Inset, high magnification. **B** Kaplan–Meier curve estimates of mice implanted with xenograft non-forming YMG25P (blue) and forming YMG25R (red).** C** Sanger sequencing indicating *IDH2* (c.515G > A, R172K) and *TP53* (c.742C > T, R248W) mutation in YMG25R xenograft. **D** Unsupervised clustering using t-SNE analysis for YMG25R xenograft. Brain tumor classifier was determined by v11b4. **E** Copy number profiling of YMG25R xenograft. **F** Immunohistochemistry for indicated proteins in YMG25R xenograft (upper) and sham mouse brain (lower). Bars, 50 μm

To assess the potential of xenograft formation, we attempted to establish orthotopic patient-derived xenograft models. YMG25P and YMG25R cells (1 × 10^5^ cells) were stereotactically injected into SCID-Beige mouse brains. Of note, we observed reproducible xenograft formation in YMG25R-implanted mice, but not in YMG25P-implanted mice (Fig. 4A, B). Hematoxylin and eosin staining of YMG25R xenografts showed proliferative tumor cells with nuclear atypia (Fig. 4A). Immunohistochemical analysis demonstrated expression of p53 and Ki-67 labeling index was 20% (Additional file 1: Fig. S4A). We found the *IDH2*^*R172K*^ and *TP53*^*R248W*^ heterozygous SNVs in the xenografts, retained from the patient tumors (Fig. 4C). Methylation classifier analysis (11b4) indicated classification matched to diffuse glioma, IDH mutant (calibrated score 0.91); subclass high grade astrocytoma (score 0.68, Fig. 4D, Additional file 2: Table S4), mirroring the parent tumor specimen. Consistent with YMG25R parent tumor cells, gain of *PDGFRA* and amplification of *CDK4* and *MDM2* were observed, while *CDKN2A* HD was identified in the xenografts, qualifying for astrocytoma, IDH-mutant, CNS WHO grade 4 according to the WHO CNS5 criteria (Fig. 4E, Additional file 1: Fig. S4B). In addition, similar to YMG25R parent tumor (Fig. 3A-B), YMG25R xenograft cells highly expressed phospho-PDGFRA, -AKT, and -ERK as well as CDK4 and MDM2, and phospho-Rb, as compared to sham control (Fig. 4F, Additional file 1: Fig. S4C–D).

To verify if *IDH1/2*-mutant astrocytomas with *CDKN2A*, *PDGFRA*, *CDK4*, or *MDM2* CNA promotes poor prognosis, we used the GLASS and MSK diffuse glioma datasets (total 1,448 cases) [6, 19]. In these datasets, we found only one case of *IDH2*-mutant and 1p/19q non-codel tumor, but CNA was not analyzed in this case. In *IDH1*-mutant astrocytoma cases with available clinical and CNA data (total 161 cases), we found that tumors harboring either *CDKN2A* deletion, *PDGFRA* amplification, *CDK4* amplification, or *MDM2* amplification conferred poor prognosis in these cohorts (Additional file 1: Fig. S5A-B).

## Discussion

In this report, we demonstrate the first novel *IDH2*-mutant patient-derived xenograft model established from a progressed recurrent astrocytoma, IDH2^R172K^ mutant, CNS WHO grade 3. Although the recurrent tumor was clinically aggressive and lethal, acquired pathogenic SNV was not annotated. Notably, we found that xenografts only formed from the recurrent tumor harboring the gain of *PDGFRA* and amplification of *CDK4* and *MDM2*, which were not identified in the initial tumor. We also found highly expressed phospho-PDGFRA and phospho-Rb in the recurrent tumor. Importantly, we confirmed that *PDGFRA*, *CDK4*, and *MDM2* CNAs as well as *IDH2*^*R172K*^ and *TP53*^*R248W*^ SNVs were recapitulated in the xenograft model. On the other hand, hemizygous deletion of *CDKN2A/B* observed in both initial and recurrent parent tumors was changed to HD in the xenograft model. These findings suggest that, similar to *IDH1*-mutant astrocytoma, co-existing CNAs that activate retinoblastoma (RB) and PDGFR signaling pathway may critically drive tumor progression and xenograft formation in *IDH2*-mutant astrocytoma.

In the GLASS and MSK diffuse glioma datasets, we found only one *IDH2*-mutant astrocytic tumor in the entire cohort [6, 19]. In addition, all 4 *IDH2*-mutant and 1p/19q non-codel tumors were histologically diagnosed as WHO grade 2 and did not show putative driver SNV, except *TP53* and *ATRX* mutations in the TCGA LGG cohort [9]. On the other hand, the present case harbored *IDH2*^*R172K*^ and *TP53*^*R248W*^ heterozygous mutation and the DNA methylation array indicated methylation class family glioma, IDH mutant, subclass astrocytoma in primary tumor and high-grade astrocytoma in recurrent tumor. Thus, this case is particularly unique and useful for better understanding molecular mechanisms of tumor progression in *IDH2*-mutant astrocytoma.

In *IDH*-mutant astrocytoma, total CNA level was associated with poor prognosis [3, 26]. One of the most critical CNAs that drive tumor progression is *CDKN2A/B* loss. In normal cells, cell cycle regulation is critical for homeostasis. *CDKN2A* encodes p14ARF and p16INK4a, while *CDKN2B* encodes p15INK4b tumor suppressor proteins. In unstressed conditions, p16INK4a and p15INK4b bind to CDK4/CDK6, while p14ARF negatively regulates MDM2, which blocks p53 accumulation. These tumor suppressor proteins block cell cycle transition from G1 phase to S phase and induce cell cycle arrest [15]. Conversely, *CDKN2A/B* deletion inactivates p16INK4a, p14ARF, and p15INK4b, deregulates cell cycle and increases cell proliferation [36]. *CDKN2A/B* HD has been demonstrated to be strongly associated with poor prognosis in *IDH*-mutant astrocytomas [37], and the WHO CNS5 defines *IDH*-mutant astrocytomas with *CDKN2A/B* HD as CNS WHO grade 4, regardless of histological findings [8, 15, 22]. In addition to *CDKN2A* HD, recent studies indicated that *CDKN2A* hemizygous deletion also confers worse prognosis in *IDH*-mutant astrocytoma [18, 21]. In the present case, both initial (YMG25P) and recurrent (YMG25R) tumors were diagnosed as WHO grade 3, because of the lack of histopathological grade 4 features (i.e., necrosis and/or microvascular proliferation) and *CDKN2A/B* HD. Notably, we found *CDKN2A/B* HD in the xenograft model (YMG25R-PDX), which was only derived from the recurrent tumor. This implies that most malignant subclonal population with *CDKN2A/B* HD may have selectively generated the xenografts. However, verifying this hypothesis will require an assay system that, unlike MLPA, enables distinguishing subclonal HD from hemizygous deletion. Besides, we found *CDK4* and *MDM2* amplifications with upregulated phospho-Rb in the recurrent tumor and its xenografts, suggesting the multiple deregulated cell cycle mechanisms to support tumor progression and facilitate xenograft formation in the present case.

Although the overall prognostic significance is still controversial [37], a large-scale study demonstrated that in addition to *CDKN2A/B* deletion, *CDK4* amplification, which also deregulates Rb pathway, was associated with poor prognosis in *IDH*-mutant astrocytoma [3, 27]. Another study demonstrated that combination of *CDK4* amplification and/or *CDKN2A* deletion, and chromosome 14 loss conferred poor prognosis in astrocytoma, IDH-mutant [10, 11]. Moreover, co-amplification of *CDK4* and *MDM2*, which are located at the breakpoint-enriched region of chromosome 12q14-15, have been previously correlated with worse clinical prognosis in GBM [49]. In the present case, co-copy number amplification of *CDK4* and *MDM2* may have cooperatively upregulated cell cycle and promoted tumor progression. In other words, analogous to *CDKN2A/B* HD, *CDK4* and *MDM2* co-amplification might accelerate tumor progression and may induce a malignant phenotype. Of note, we found that CDK4/6 inhibitor selectively suppressed cell viability of phospho-Rb-upregulated, recurrent cells, further supporting the critical role of cell cycle deregulation in progression in *IDH2*-mutant astrocytoma.

We also found *PDGFRA* CNA in the recurrent tumor as well as the xenograft model and upregulated protein expressions in the PI3K/AKT/mTOR pathway and RAS/RAF/MEK/ERK pathway in the recurrent tumor. It has been demonstrated that *PDGFRA* amplification is correlated with *IDH1* mutation and associated with poorer prognosis in *IDH1*-mutant astrocytomas, CNS WHO grade 4, as compared to those without *PDGFRA* amplification [32]. Yang et al. stratified *IDH*-mutant lower-grade astrocytomas by the presence of *CDKN2A* HD, *CDK4* amplification, and *PDGFRA* amplification. These copy number alterations were found in a mutually exclusive manner [47], unlike the present case. They found that tumors with *PDGFRA* amplification (high risk group) had poorer prognosis than those with *CDKN2A* homozygous deletion or *CDK4* amplification (intermediate group) and copy number non-altered group (low risk group) [47]. *PDFGRA* is a member of the receptor tyrosine kinase family and is involved in stimulating the PI3K/AKT/mTOR pathway and RAS/RAF/MEK/ERK pathway [7]. *PDGFRA* plays a role in normal gliogenesis of the central nervous system and *PDGFRA* high-level amplification and gain has been associated with high grade malignancy in gliomas [1, 34]. In an experimental model, PDGFA enhanced the growth of IDH1^R132H^ mutant immortal *Cdkn2a*, *Atrx*, and *Pten* deficient astrocytes and PDGFA cooperated with IDH1^R132H^ and loss of *Cdkn2a*, *Atrx*, and *Pten* to promote glioma formation in vivo [31]. Another study using the RCAS/TVA system demonstrated that glioma-genesis in the context of mutant IDH1 with shp53 and/or *Cdkn2a* loss, was only facilitated when combined with PDGFa [2]. Importantly, the present data demonstrated that PDGFR inhibitor potently suppressed cell viability in recurrent tumor cells with *PDGFRA* gain, whereas there was no difference after PI3K inhibitor treatment. These clinical and preclinical findings support the role of *PDGFRA* gene alterations and downstream multiple signaling pathways, including PI3K/AKT/mTOR pathway and RAS/RAF/MEK/ERK pathway, in promoting progression of not only *IDH1*-mutant, but also *IDH2*-mutant astrocytoma.

Although a recent clinical trial reported that an inhibitor targeting mutant *IDH1* and *IDH2* induced durable therapeutic efficacy in lowgrade glioma [24], the impact of directly targeting mutant *IDH* for high-grade glioma is under clinical investigation. In accord with our previous study that IDH1 inhibitor was not sufficient to induce anti-tumor effects in *IDH1*-mutant high-grade gliomas [40], we did not find decreased cell viability nor histone methylation status changes after mutation specific IDH2 inhibitor treatment of recurrent cells. However, as the drug exposure was short-term, further study is needed to address if prolonged use of mutant specific IDH2 inhibitor can induce anti-tumor effects in IDH2 mutant high-grade astrocytoma. Impact of IDH2 inhibitors on earlier stages of tumorigenesis also requires future investigations.

Altogether, this study demonstrated the pivotal biological role of gene alterations that activate RB pathway and PDGFR pathway in the progression of *IDH2*-mutant astrocytoma. This molecular mechanism in disease progression seems analogous to *IDH1*-mutant astrocytoma. We also established the first *IDH2-*mutant astrocytoma xenograft model derived from progressed disease. Together with the clinical characteristics and xenograft model, we found that CNAs involving RB pathway and *PDGFRA* promote tumor progression in astrocytoma, IDH2-mutant.

### Supplementary Information

**Additional file 1. Figure S1.** **A** Positron emission tomography indicated uptake (arrow head) of 18F-FDG (left) and 11C-methionine (right) in initial tumor (YMG25P). **B** Immunohistochemistry for indicated proteins in YMG25P and YMG25R tumor. Bars, 50μm; **Figure S2.** **A** Multiplex ligation-dependent probe amplification (MLPA) indicating copy number alterations for indicated genes in YMG25P (upper) and YMG25R (lower). **B** MLPA indicating chromosome partial deletion of 19q, CDKN2A hemizygous loss, and IDH2R172K mutation in YMG25P (upper). MLPA indicating chromosome partial deletion of 1p and 19q, CDKN2Ahemizygous loss, and IDH2R172K mutation in YMG25R (lower). **C** DNA methylation array indicating MGMT promotor methylation status in initial tumor (YMG25P, left) and recurrent tumor (YMG25R, right); **Figure S3.** **A** Immunohistochemistry for indicated proteins in initial (YMG25P, upper) and recurrent tumors (YMG25R, lower). **B** Western blotting of indicated proteins in YMG25P and YMG25R tumors. Bars, 50μm. **C** Relative cell viability of PI3K inhibitor (LY294002) and AKT inhibitor (GDC-0068) at day3. **D** Relative cell viability of YMG25R cells after IDH2 inhibitor (AG-221) at day 9. DMSO, control. Data are represented as the mean ± SEM. **E**, **F** Western blotting of indicated proteins in YMG25R cells after DMSO or AG-221 (5μM) treatment for 12 days. NS, not significant; **Figure S4.** **A** Immunohistochemistry for indicated proteins in YMG25R xenograft tumor. **B** Multiplex ligation-dependent probe amplification indicating copy number alterations for indicated genes in YMG25R xenograft. **C** Immunohistochemistry for indicated proteins in YMG25R xenograft (upper) and sham mouse brain (lower). Bars, 50μm. **D** Bar graphs indicating % immuno-positive cells for indicated proteins. *P < 0.05; **Figure S5.** **A** Genomic landscape of IDH-mutant astrocytoma with/without copy number alterations. GLASS and MSK lower-grade glioma cohorts are merged for analysis. **B** Kaplan-Meier curve showing survival difference of IDH1-mutant astrocytoma with/without either PDGFRAamplification, CDK4 amplification, MDM2 amplification, or CDKN2A deletion. *P*-value is determined by Log-rank test.

**Additional file 2.** **Table S1.** Primers used for PCR amplification and sequencing; **Table S2.** Whole exome sequencing for initial tumor (YMG25P); **Table S3.** Whole exome sequencing for recurrent tumor (YMG25R); **Table S4.** DNA methylation array-based tumor classification (version 11b4); **Table S5:** DNA methylation array-based tumor classification (version 12.8).

## Data Availability

The genomic and epigenomic information used in this study was deposited in the National Bioscience Database Center under accession number JSUB000906. The data generated in this study are available within the article and the online supplementary material.
