# Supplementary material for: Genetic alterations that deregulate RB and PDGFRA signaling pathways drive tumor progression in IDH2-mutant astrocytoma
Source: Acta Neuropathol Commun. 2023 Nov 27;11:186. doi: 10.1186/s40478-023-01683-x (PMC10680361; doi:10.1186/s40478-023-01683-x)
Supplement: Supplementary file 1 — Additional file 1. Figure S1. A Positron emission tomography indicated uptake (arrow head) of 18F-FDG (left) and 11C-methionine (right) in initial tumor (YMG25P). B Immunohistochemistry for indicated proteins in YMG25P and YMG25R tumor. Bars, 50μm; Figure S2. A Multiplex ligation-dependent probe amplification (MLPA) indicating copy number alterations for indicated genes in YMG25P (upper) and YMG25R (lower). B MLPA indicating chromosome partial deletion of 19q, CDKN2A hemizygous loss, and IDH2R172K mutation in YMG25P (upper). MLPA indicating chromosome partial deletion of 1p and 19q, CDKN2Ahemizygous loss, and IDH2R172K mutation in YMG25R (lower). C DNA methylation array indicating MGMT promotor methylation status in initial tumor (YMG25P, left) and recurrent tumor (YMG25R, right); Figure S3. A Immunohistochemistry for indicated proteins in initial (YMG25P, upper) and recurrent tumors (YMG25R, lower). B Western blotting of indicated proteins in YMG25P and YMG25R tumors. Bars, 50μm. C Relative cell viability of PI3K inhibitor (LY294002) and AKT inhibitor (GDC-0068) at day3. D Relative cell viability of YMG25R cells after IDH2 inhibitor (AG-221) at day 9. DMSO, control. Data are represented as the mean ± SEM. E, F Western blotting of indicated proteins in YMG25R cells after DMSO or AG-221 (5μM) treatment for 12 days. NS, not significant; Figure S4. A Immunohistochemistry for indicated proteins in YMG25R xenograft tumor. B Multiplex ligation-dependent probe amplification indicating copy number alterations for indicated genes in YMG25R xenograft. C Immunohistochemistry for indicated proteins in YMG25R xenograft (upper) and sham mouse brain (lower). Bars, 50μm. D Bar graphs indicating % immuno-positive cells for indicated proteins. *P < 0.05; Figure S5. A Genomic landscape of IDH-mutant astrocytoma with/without copy number alterations. GLASS and MSK lower-grade glioma cohorts are merged for analysis. B Kaplan-Meier curve showing survival difference of IDH1-mutant astrocyt [file 40478_2023_1683_MOESM1_ESM.pdf]

Supplementary Figure 1

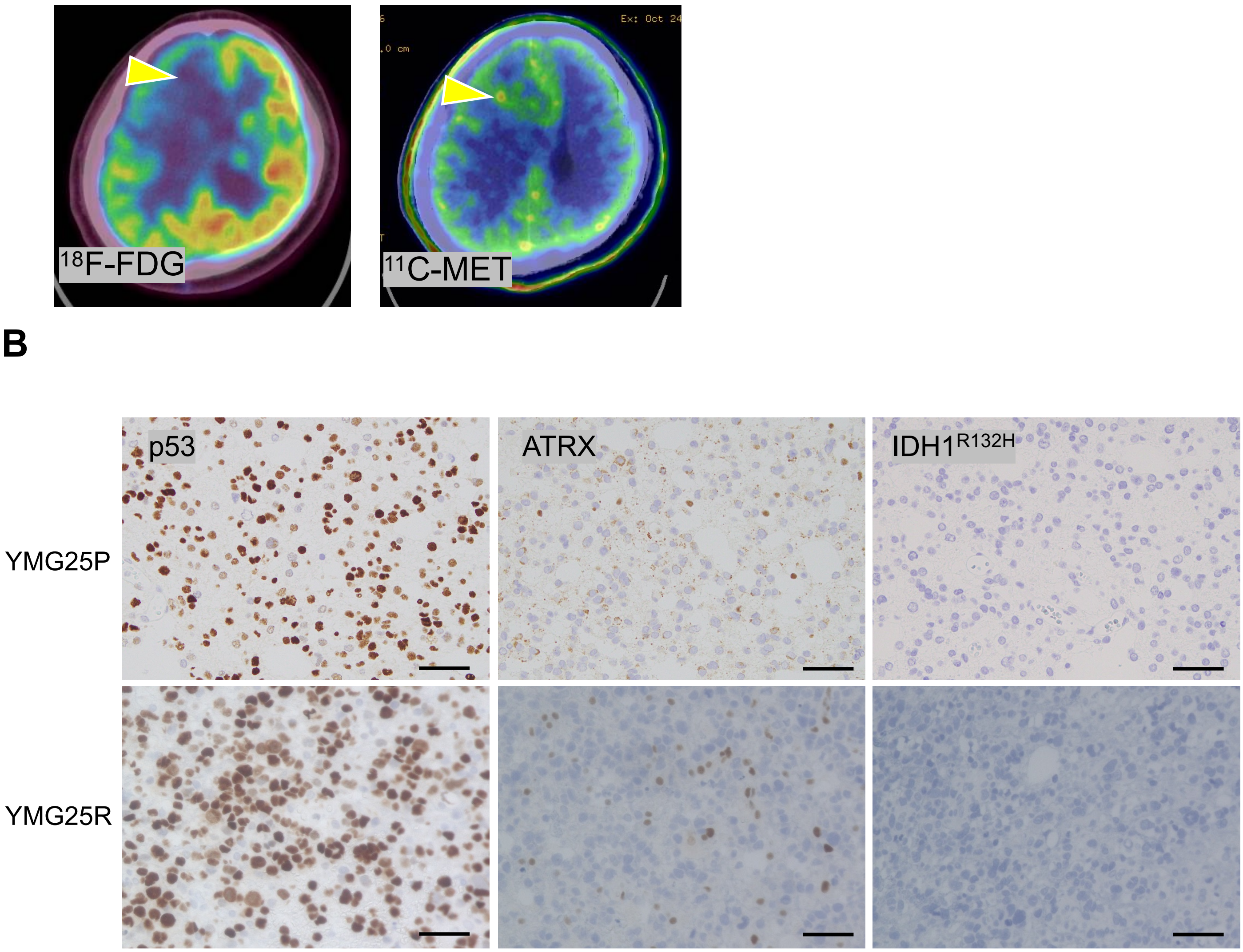

Supplementary Figure 1. A, Positron emission tomography indicated uptake (arrow head) of ^{18}F-FDG (left) and ^{11}C-methionine (right) in initial tumor (YMG25P). **B**, Immunohistochemistry for indicated proteins in YMG25P and YMG25R tumor. Bars, 50μm.

Supplementary Figure 2

A

YMG25P

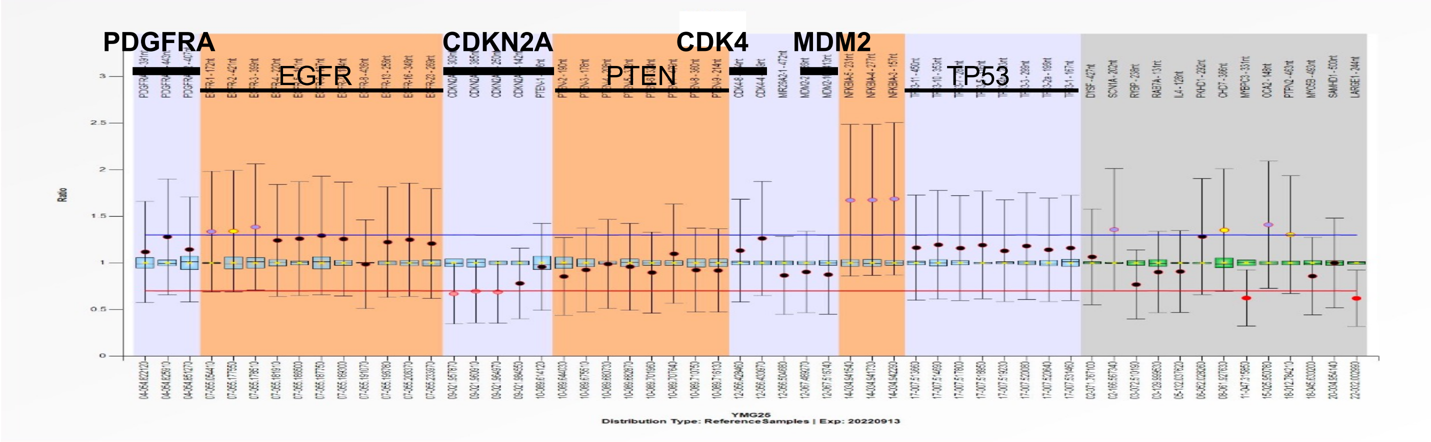

YMG25R

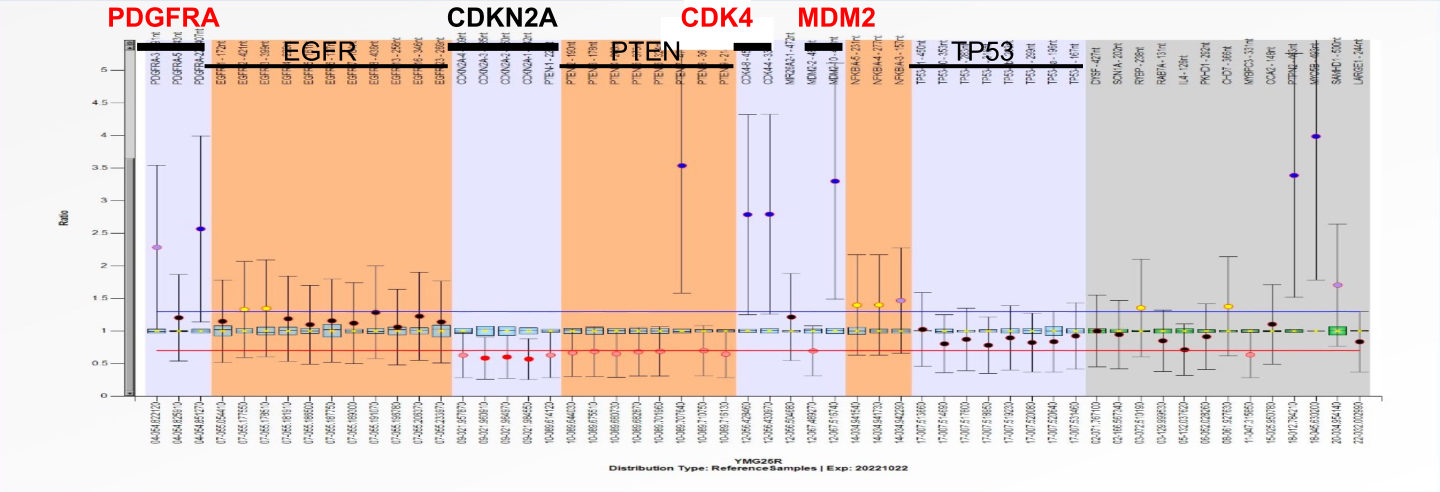

Supplementary Figure 2 (continued)

B

YMG25P

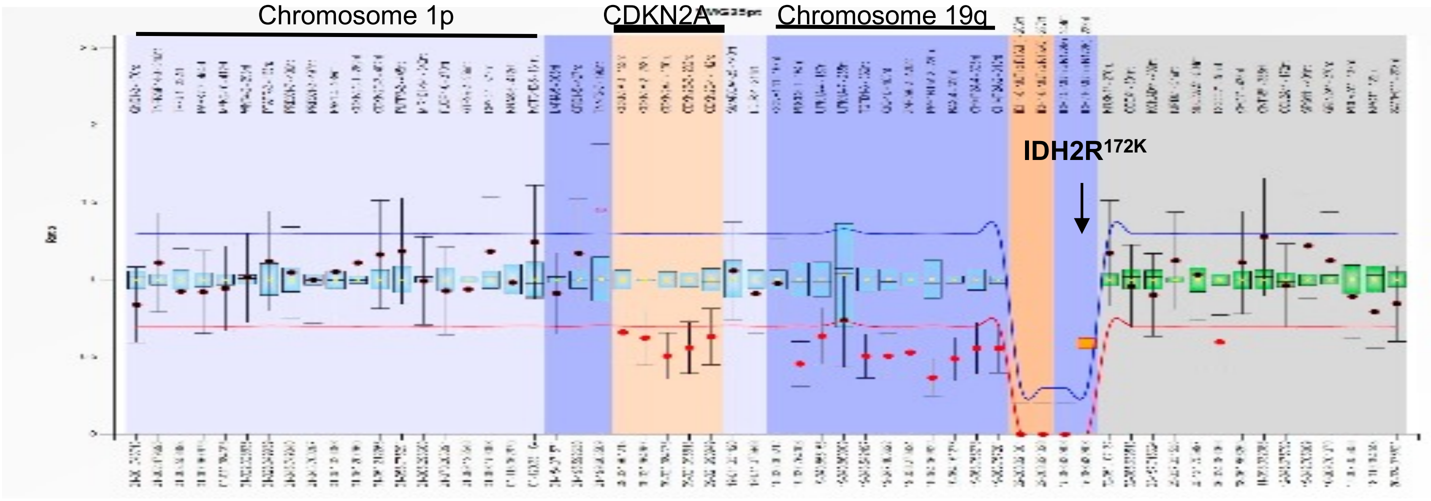

YMG25R

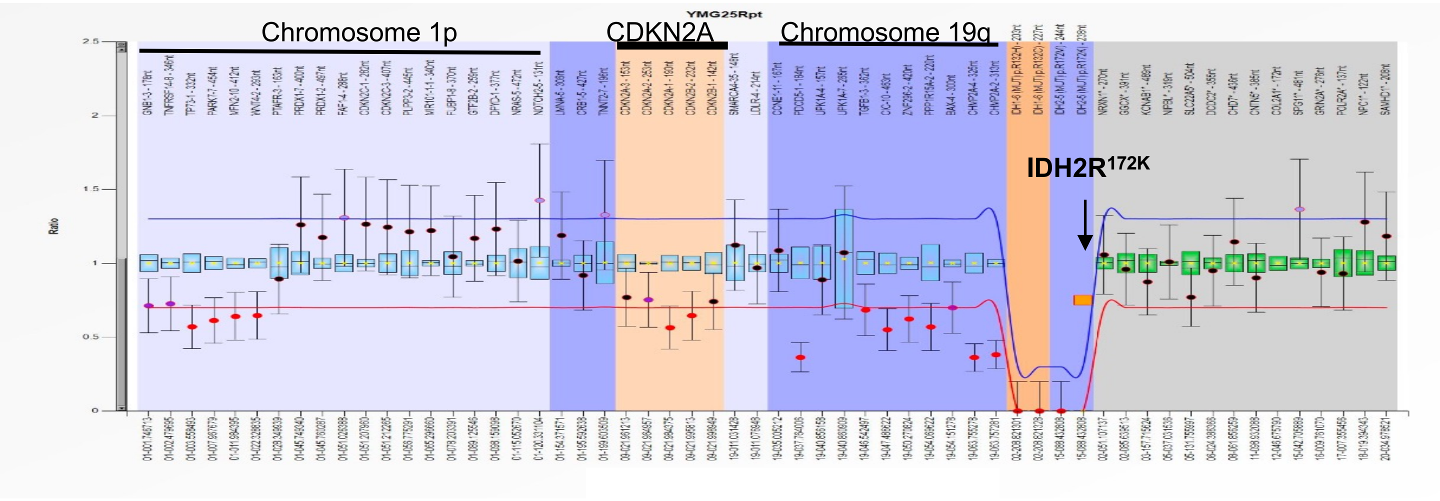

C

YMG25P

MGMT promotor status prediction

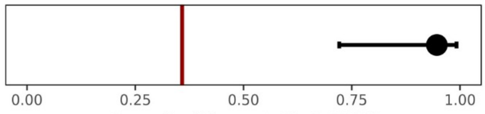

YMG25R

MGMT promotor status prediction

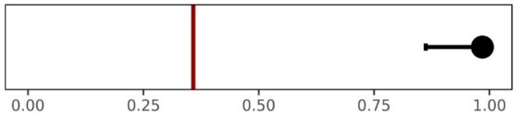

Supplementary Figure 2. A Multiplex ligation-dependent probe amplification (MLPA) indicating copy number alterations for indicated genes in YMG25P (upper) and YMG25R (lower). **B**, MLPA indicating chromosome partial deletion of 19q, *CDKN2A* hemizygous loss, and *IDH2*^{R172K} mutation in YMG25P (upper). MLPA indicating chromosome partial deletion of 1p and 19q, *CDKN2A* hemizygous loss, and *IDH2*^{R172K} mutation in YMG25R (lower). **C**, DNA methylation array indicating *MGMT* promotor methylation status in initial tumor (YMG25P, left) and recurrent tumor (YMG25R, right).

Supplementary Figure 3

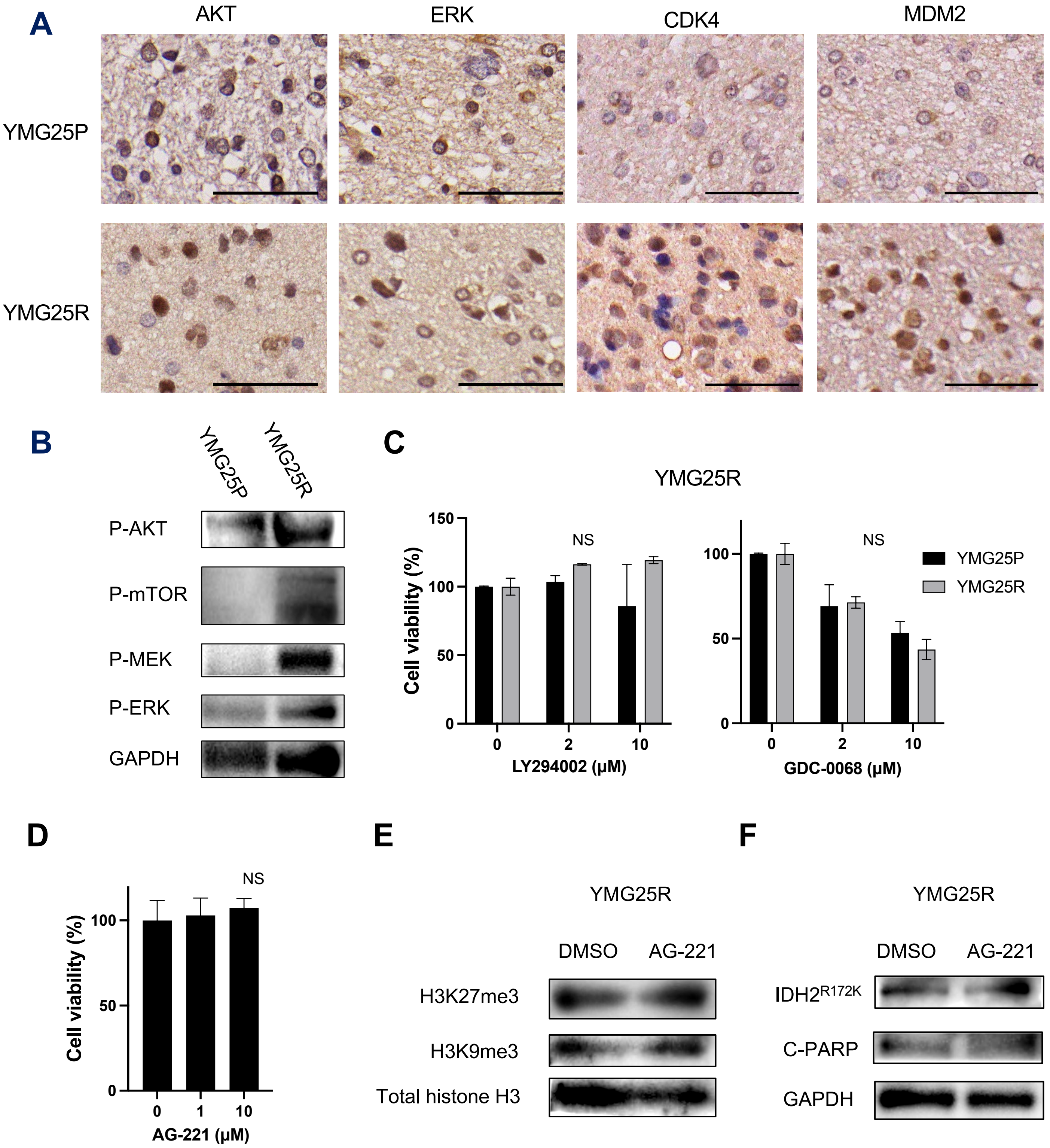

Supplementary Figure 3. A, Immunohistochemistry for indicated proteins in initial (YMG25P, upper) and recurrent tumors (YMG25R, lower). **B**, Western blotting of indicated proteins in YMG25P and YMG25R tumors. Bars, 50μm. **C**, Relative cell viability of PI3K inhibitor (LY294002) and AKT inhibitor (GDC-0068) at day3. **D**, Relative cell viability of YMG25R cells after IDH2 inhibitor (AG-221) at day 9. DMSO, control. Data are represented as the mean ± SEM. **E, F,** Western blotting of indicated proteins in YMG25R cells after DMSO or AG-221 (5μM) treatment for 12 days. NS, not significant.

Supplementary Figure 4

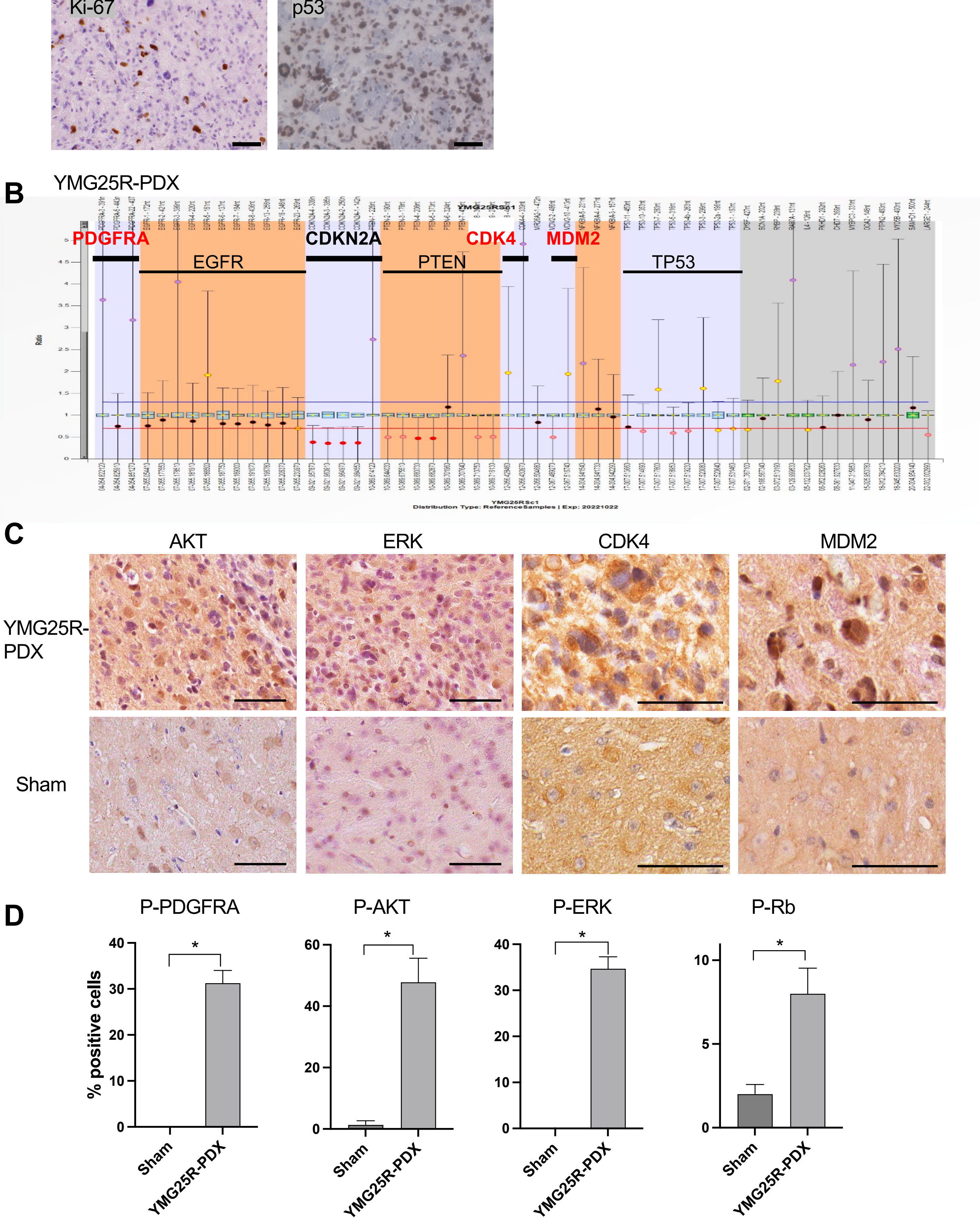

Supplementary Figure 4. A, Immunohistochemistry for indicated proteins in YMG25R xenograft tumor. **B,** Multiplex ligation-dependent probe amplification indicating copy numbe alterations for indicated genes in YMG25R xenograft. **C,** Immunohistochemistry for indicate proteins in YMG25R xenograft (upper) and sham mouse brain (lower). Bars, 50μm. **D,** Bar graphs indicating % immuno-positive cells for indicated proteins. *, $P < 0.05$.

Supplementary Figure 5

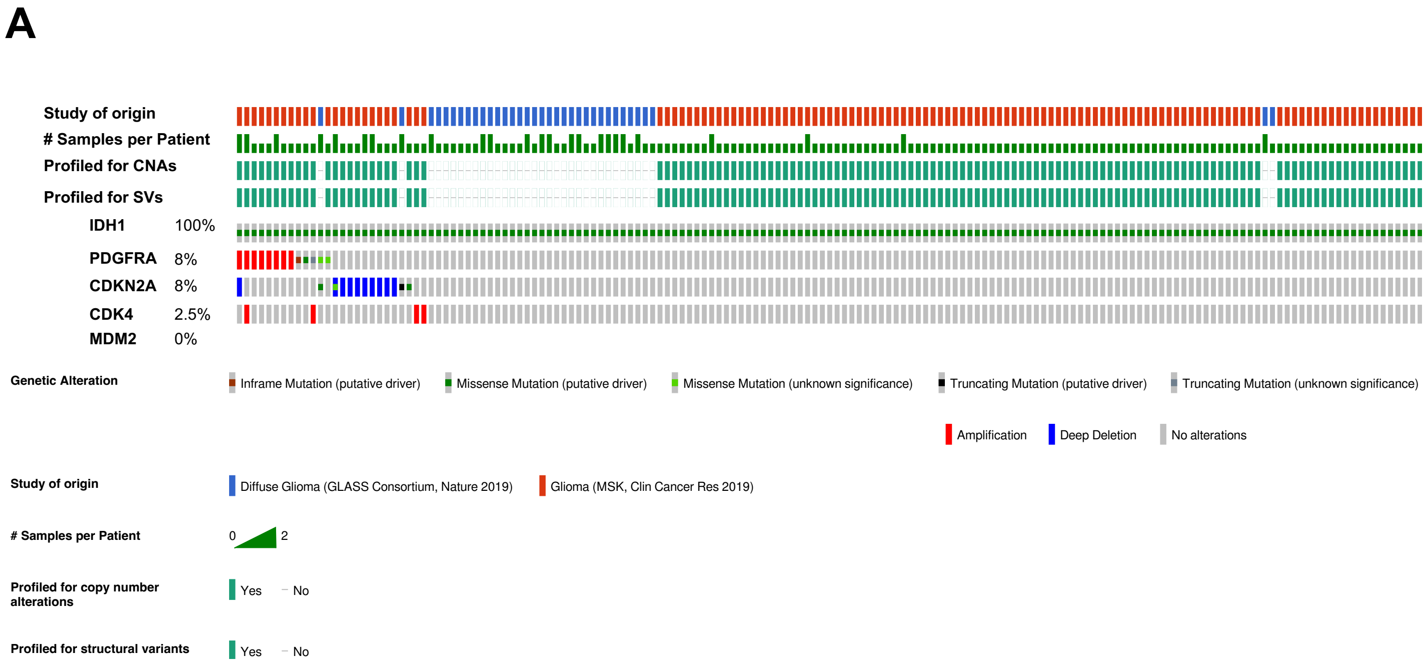

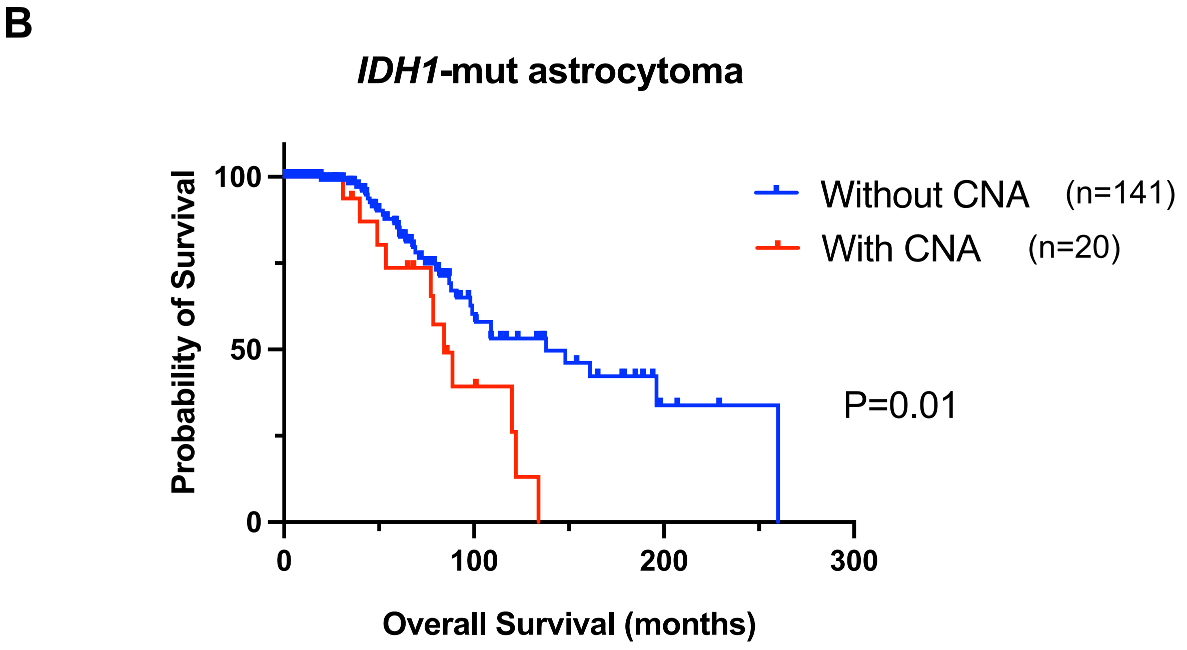

Supplementary Figure 5. A, Genomic landscape of *IDH*-mutant astrocytoma with/without copy number alterations. GLASS and MSK lower-grade glioma cohorts are merged for analysis. **B,** Kaplan-Meier curve showing survival difference of *IDH1*-mutant astrocytoma with/without either *PDGFRA* amplification, *CDK4* amplification, *MDM2* amplification, or *CDKN2A* deletion. *P*-value is determined by Log-rank test.
